# Electron Donation Stabilizes Pt Catalysts in Methanol Fuel Cells

**DOI:** 10.1021/acscentsci.5c01712

**Published:** 2025-09-20

**Authors:** Xin Wan, Jianglan Shui

**Affiliations:** † School of Materials Science and Engineering, Beihang University, Beijing 100191, China; ‡ School of Space and Earth Sciences, Beihang University, Beijing 102206, China; § School of Materials Science and Engineering, Beihang University, Beijing 100191, China; ⊥ Tianmushan Laboratory, Hangzhou 310023, China

## Abstract

An electron-enriched PtNiCo catalyst enabled by TiN support boosts stability in methanol fuel cells by simultaneously overcoming CO poisoning and metal dissolution.

## Graphical abstract

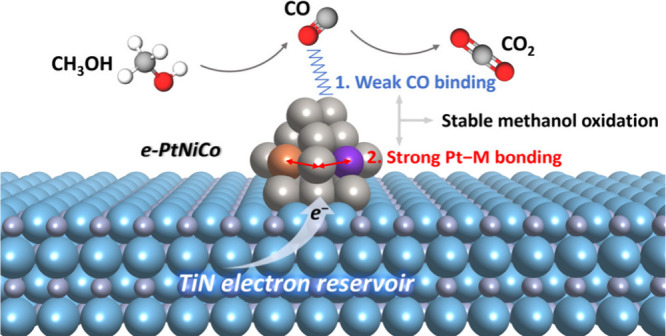

Direct methanol fuel cells (DMFCs) hold great promise for powering portable electronics and microdevices due to the high energy density and facile storage of liquid methanol. However, their commercialization is limited by the sluggish kinetics of the methanol oxidation reaction (MOR) at the anode. This multielectron process not only generates carbon dioxide but also inevitably produces carbon monoxide (CO), which is a poisonous intermediate.^1^ Carbon-supported Pt-based catalysts, particularly those alloyed with transition metals such as Ru, Ni, or Co, are widely used to improve activity and mitigate CO poisoning. Yet, under realistic DMFC conditions (high methanol concentrations, elevated temperatures, and acidic environment), these catalysts still face a dual threat: strong chemisorption of CO that blocks Pt active sites and continuous dissolution of the nonprecious alloying metals, which degrades catalytic performance and damages the fuel cell membrane.^2,3^ Currently, the rational design of Pt-alloy catalysts with dual resistance to CO poisoning and metal leaching remains a critical challenge.

Currently, the rational design of Pt-alloy catalysts with dual resistance to CO poisoning and metal leaching remains a critical challenge.

In this issue of *ACS Central Science*, Tian, Miao, and co-workers report a compelling solution.^4^ They present an electron-enriched PtNiCo catalyst anchored on titanium nitride (TiN), denoted as e-PtNiCo, which achieves remarkable stability by simultaneously suppressing CO poisoning and metal dissolution. Utilizing strong metal–support interactions between Pt and noncarbon supports offers an effective strategy to modulate the *d*-band center, a key descriptor of CO adsorption strength.^5–7^ Previous efforts have explored metal oxides and carbides for creating electron-modified interfaces, but these still degrade under operational conditions.^6,7^ In contrast, TiN combines high electrical conductivity with exceptional corrosion resistance.^8–10^ The ingenuity of this work lies in leveraging TiN not only as a robust and conductive support but also as an electron reservoir that continuously donates charge to the Pt alloy. This fundamentally alters the electronic structure of Pt atoms, reshaping their interactions with both adsorbed CO species and neighboring alloy metal atoms.

Through X-ray photoelectron spectroscopy (XPS) and X-ray absorption spectroscopy (XAS), the authors confirm a lower valence state of Pt in e-PtNiCo compared to its carbon-supported counterparts, indicating substantial electron transfer from TiN to the alloy. Density functional theory (DFT) calculations quantify this transfer as 3.51 *e* ^–^, leading to an electron-enriched catalytic interface. As illustrated in Figure 1, this electron enrichment yields two profound effects. First, it downshifts the *d*-band center of surface Pt atoms, significantly weakening CO adsorption (with the adsorption free energy reduced from −1.62 eV to −1.27 eV), thereby facilitating its oxidative removal at lower potentials. Second, it strengthens the metallic bonds within the alloy (Pt–Ni, Pt–Co), making the structure more resistant to acidic dissolution.

**Figure 1:**
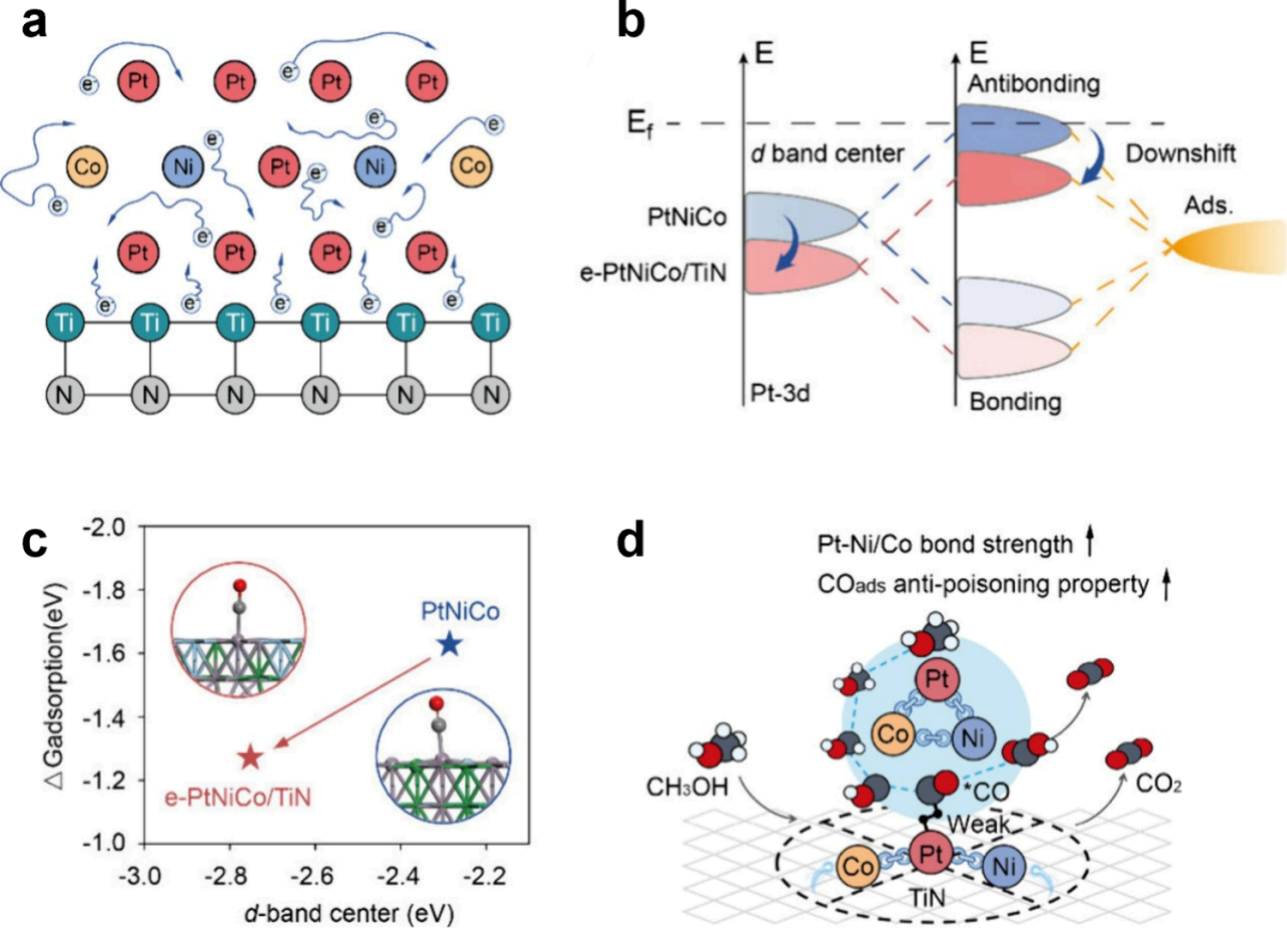
(a) The charge transfer model for e-PtNiCo. (b) Schematic illustration of the *d*-band position of e-PtNiCo. (c) Relationship between the *d*-band center of Pt atoms and CO adsorption free energy. (d) Dual modulation diagram of the electron enrichment. Reproduced with permission from ref 4. Available under a CC-BY 4.0 license. Copyright 2025 Min Chen, Yichi Guan, Zhengpei Miao, Shuo Zhang, Chunxia Wu, Yu Zhou, Hongxian Luo et al.

The ingenuity of this work lies in leveraging TiN not only as a robust and conductive support but also as an electron reservoir that continuously donates charge to the Pt alloy.

The electron-enriched catalyst demonstrates outstanding electrochemical performance. In half-cell tests, e-PtNiCo displays a lower onset potential for methanol oxidation (0.50 V vs RHE) and achieves a mass activity of 1.73 A mg_Pt_^–1^, outperforming all control catalysts. It also shows the lowest onset potential for oxidizing adsorbed CO intermediates, indicating a favorable anti-CO poisoning ability. After 5000 accelerated durability test cycles, e-PtNiCo retains 83.8% of its initial activity, significantly exceeding that of PtNiCo (56.8%) and Pt/C (47.3%). This superior durability is attributed to a 2-fold reduction in Ni/Co dissolution and enhanced structural integrity. More importantly, in a practical DMFC system, e-PtNiCo delivers a peak power density of 107 mW cm^–2^ at 65 °C and maintains 90.4% of its initial voltage after 50 h of operation at 100 mA cm^–2^, representing a 4-fold improvement over the carbon-supported PtNiCo catalyst.

This work establishes a smart strategy for stabilizing Pt-based catalysts using an electron-donating support. By elucidating the dual mechanisms of antipoisoning and dissolution inhibition, Tian, Miao, and co-workers provide a blueprint for designing durable electrocatalysts. Their adoption of TiN as an electron reservoir may inspire catalyst design across diverse electrochemical energy technologies. Future studies might explore other conductive metal nitrides or fine-tune the degree of electron donation to further optimize performance.

## References

[1] Wang J., Zhang B., Guo W., Wang L., Chen J., Pan H., Sun W. (2023). Toward Electrocatalytic
Methanol Oxidation Reaction:
Longstanding Debates and Emerging Catalysts. Adv. Mater..

[2] Wang L., Luo L., Guo Z., Wang Y., Liu X. (2025). Challenges and strategic
advancements in platinum-based catalysts for tailored methanol oxidation
reaction. J. Electroanal. Chem..

[3] Liu W., Liu D., Wan X., Shui J. (2025). Functional additives for proton exchange
membrane fuel cells. EnergyChem..

[4] Chen M., Guan Y., Miao Z., Zhang S., Wu C., Zhou Y., Luo H., Wu D., Li R., Luo J., Tian X. (2025). Revealing the Stabilization
Mechanism of Electron-Enriched
PtNiCo Catalysts in Practical Direct Methanol Fuel Cells. ACS Cent. Sci..

[5] Xu A., Liu T., Liu D., Li W., Huang H., Wang S., Xu L., Liu X., Jiang S., Chen Y., Sun M., Luo Q., Ding T., Yao T. (2024). Edge-Rich Pt–O–Ce Sites
in CeO_2_ Supported Patchy Atomic-Layer Pt Enable a Non-CO
Pathway for Efficient Methanol Oxidation. Angew.
Chem., Int. Ed..

[6] Li S., Wang G., Lv H., Lin Z., Liang J., Liu X., Wang Y. G., Huang Y., Wang G., Li Q. (2024). Constructing
Gradient Orbital Coupling to Induce Reactive Metal-Support Interaction
in Pt-Carbide Electrocatalysts for Efficient Methanol Oxidation. J. Am. Chem. Soc..

[7] Mao G., Zhou Q., Wang B., Xiong Y., Zheng X., Ma J., Fu L., Luo L., Wang Q. (2025). Modulating d-Orbital
electronic configuration via metal-metal oxide interactions for boosting
electrocatalytic methanol oxidation. J. Colloid
Interface Sci..

[8] Lee J.-M., Han S.-B., Song Y.-J., Kim J.-Y., Roh B., Hwang I., Choi W., Park K.-W. (2010). Methanol electrooxidation
of Pt catalyst on titanium nitride nanostructured support. Appl. Catal. A-Gen..

[9] Xiao Y., Zhan G., Fu Z., Pan Z., Xiao C., Wu S., Chen C., Hu G., Wei Z. (2014). Robust non-carbon titanium
nitride nanotubes supported Pt catalyst with enhanced catalytic activity
and durability for methanol oxidation reaction. Electrochim. Acta.

[10] Tang M., Ding H., Zhang Y., Long Y., Liu J., Jin M., Yi H., Luo L., Lu S.-Y., Zhang J. (2024). Pt Nanoparticles
Supported on N-Doped Carbon/Mesoporous TiN Particle Composites as
Catalysts for the Methanol Oxidation Reaction. ACS Appl. Nano Mater..

